# Efficient Adsorption of Lead (II) from Aqueous Phase Solutions Using Polypyrrole-Based Activated Carbon

**DOI:** 10.3390/ma12122020

**Published:** 2019-06-24

**Authors:** Abdulaziz Ali Alghamdi, Abdel-Basit Al-Odayni, Waseem Sharaf Saeed, Abdullah Al-Kahtani, Fahad A. Alharthi, Taieb Aouak

**Affiliations:** Chemistry Department, College of Science, King Saud University, P.O. Box 2455, Riyadh 11451, Saudi Arabia; aalghamdia@ksu.edu.sa (A.A.A.); akahtani@ksu.edu.sa (A.A.-K.); fharthi@ksu.edu.sa (F.A.A.); taouak@ksu.edu.sa (T.A.)

**Keywords:** polypyrrole, polypyrrole-based activated carbon, water pollution, heavy metal removal, lead ions, adsorption

## Abstract

In this study, polypyrrole-based activated carbon was prepared by the carbonization of polypyrrole at 650 °C for 2 h in the presence of four-times the mass of KOH as a chemical activator. The structural and morphological properties of the product (polypyrrole-based activated carbon (PPyAC4)), analyzed by scanning electron microscopy, transmission electron microscopy, X-ray diffraction, and thermogravimetric analysis, support its applicability as an adsorbent. The adsorption characteristics of PPyAC4 were examined through the adsorption of lead ions from aqueous solutions. The influence of various factors, including initial ion concentration, pH, contact time, and adsorbent dose, on the adsorption of Pb^2+^ was investigated to identify the optimum adsorption conditions. The experimental data fit well to the pseudo-second-order kinetic model (R^2^ = 0.9997) and the Freundlich isotherm equation (R^2^ = 0.9950), suggesting a chemisorption pathway. The adsorption capacity was found to increase with increases in time and initial concentration, while it decreased with an increase in adsorbent dose. Additionally, the highest adsorption was attained at pH 5.5. The calculated maximum capacity, *q_m_*, determined from the Langmuir model was 50 mg/g.

## 1. Introduction

Currently, water pollution is a global issue, with heavy metal pollution representing one of the most serious problems. Therefore, the removal of heavy metals is of special concern to scientists and engineers because of their harmful effects on many life forms [1,2]. Heavy metals, including lead, copper, mercury, chromium, and nickel, commonly enter aquatic systems through effluent discharge from various manufacturing processes, resulting in damages to ecosystems and human health [3,4]. They are non-biodegradable and tend to accumulate in living tissues, particularly in human bodies, causing various diseases and significant physiological disorders such as central nervous system damage [2,5]. Among all heavy metal pollutants, lead is the most common and one of the most toxic, reaching water sources from various industrial activities such as mining, oil refining, metal plating and finishing, and battery manufacturing [4,6]. The maximum contaminant level of lead in drinking water allowed by the Environmental Protection Agency (EPA) and the World Health Organization (WHO) is 15 and 50 µg/L, respectively [7]. When lead concentrations become elevated, serious health problems can occur [6]. Lead poisoning can cause kidney damage, anemia, and toxicity symptoms including impaired kidney function, hypertension, and headache [8].

Various technologies are employed for the removal of heavy metals from water including chemical precipitation [9], ion exchange [10,11], reverse osmosis [12], membrane filtration, and adsorption [11,12]. Adsorption is the most efficient and extensively-used removal method. Commonly-used adsorbents are comprised of activated carbon [1,13], zeolite [14], biomasses [15,16], polymers [17,18], and polymer composites [19,20]. However, activated carbon is one of the most effective adsorbents due to its simplicity, high capacity, and ability to remove low concentrations of lead [21,22,23]. There are two basic activation processes, physical and chemical. Physical activation requires higher temperatures compared to chemical activation processes. Chemical activation can be accomplished in one step through the thermal decomposition of raw materials in the presence of chemical reagents like acid, base, or salt. 

Although several adsorbents have been utilized for the removal of heavy metal ions from wastewater, no single adsorbent was found to be effective for different types of water pollutants [7]. Therefore, obtaining material with sufficient and desirable adsorption properties is still a challenge for scientists and engineers. Hence, polymer-based adsorbents, including conducting adsorbents such as polyaniline and polypyrrole, in addition to their composites, have received much attention owing to their potential application in the removal of heavy metals from aqueous solutions. They offer advantages such as a high surface area, a porous structure, favorable electrochemical properties, ion exchange capability, facile synthesis, simple operation and regeneration, high stability, and cost effectiveness, which favor their utilization as efficient adsorbents for the removal of water pollutants [24,25,26,27,28].

Polypyrrole powder [29] and polypyrrole-based composites composed of different materials, such as carbon nanotubes [30,31], chitosan [19], and bentonite [20], have recently been employed for the removal of heavy metal ions from aqueous solutions. In addition, the decolorizing efficiency of polypyrrole-coated sawdust has been investigated, with remarkable results [32].

Nitrogen-doped graphene oxide sheets (N-GO), which are investigated in this work, are reported to have a promising surface texture such as high surface area (2871 m^2^/g) and pore size and distribution, in addition to their excellent gas adsorption and sensing properties [33,34]. The N-GO sheets were prepared using N-containing precursors (e.g., polypyrrole) in the presence of KOH as a chemical activator and were then characterized and tested for CO_2_ capture ability. Examining the applicability of such interesting materials for the removal of water pollutants, such as heavy metals and dyes, along with other applications is needed to understand and report fully the potential of these adsorbents. 

The objective of this study is to investigate the adsorption performance of the as-prepared polypyrrole-based activated carbon (PPyAC4) using the lead ion as a model adsorbate. The effects of various parameters, including adsorbate initial concentration, pH value, and adsorbent dose, on adsorption efficiency were examined. 

## 2. Materials and Methods 

### 2.1. Materials 

Pyrrole monomer C_4_H_5_N (Py, +98%), ammonium persulfate (NH_4_)_2_S_2_O_8_ (APS, 98%), lead nitrate Pb(NO_3_)_2_ (99%), potassium hydroxide pellets KOH (85%), and sodium hydroxide pellets NaOH (98%) were purchased from Alfa Aesar, Karlsruhe, Germany. Hydrochloric acid HCl (~36%) and methanol (99.5%) were obtained from Fisher Chemical, Loughborough, U.K. All reagents were used as received without any further treatment. Deionized water was used throughout the experimental process. 

### 2.2. Preparation of PPyAC4 

The polypyrrole-based activated carbon PPyAC4 was prepared as previously reported by Alghamdi et al. [33]. Briefly, 0.04 mol of pyrrole were added to 600 mL of 0.1 M HCl and stirred for 30 min at 0–5 °C. APS (0.06 mol) was dissolved in 30 mL of deionized water, stirred for 30 min at 0–5 °C, and added dropwise to the monomer solution while stirring at 0–5 °C. The obtained solid product was filtered, washed with distilled water and methanol, and dried under reduced pressure for 48 h. The carbonization of the polypyrrole (PPy) in the presence of KOH as an activating agent was performed in a Carbolite furnace under a nitrogen atmosphere. PPy was thoroughly mixed with KOH in a 1:4 weight ratio, respectively. The mixture was heated at a ramp rate of 3 °C/min and carbonized for 2 h at 650 °C. After cooling, the product was washed several times by 0.5 M HCl, then by water to neutrality. Finally, it was dried in an oven at 100 °C overnight and then in a vacuum oven at 60 °C until a constant weight was achieved.

### 2.3. Adsorption Experiments

All adsorption experiments were carried out at room temperature (23 ± 2 °C) using 25 mL of a lead ion solution and constant agitation of 130 rpm using a digital orbital shaker (GLF 3017, GmbH, Burgwedel, Germany). For the kinetic adsorption study, 125 mg of PPyAC4 were added to the adsorbate solutions with initial concentrations of 10, 50, and 100 ppm at pH 5.5, and the residual metal ion concentration was determined at the given time intervals (0, 30, 60, 90, 120, 240, and 360 min), using a flame atomic absorption spectrophotometer (FAAS) (PinAAcle 900T, PerkinElmer, Waltham, MA, USA) with a deuterium lamp (BGC-D2) and air-acetylene flame, with a detection limit for lead ion in aqueous solutions of 0.01 mg/L. For each measurement, at least three replicates were performed and averaged. Although adsorption seemed to reach equilibrium at approximately 4 h after initial contact time, the solutions were allowed to agitate for a total period of 24 h, after which the residual Pb^2+^ concentration was measured to ensure equilibrium. The effects of pH (4.0, 5.5, and 7.0), adsorbate initial concentration (10, 50, and 100 ppm), and adsorbent dose (63, 94, and 125 mg) on the adsorption behavior were studied. For the equilibrium isotherm investigation, 125 mg of adsorbent was mixed with 25 mL of 10, 50, 100, or 250-ppm Pb^2+^ solutions at pH 5.5 with stirring for 6 h. The initial pH of the experimental solutions was adjusted to the desired pH by dropwise addition of 0.1 M HCl or 0.1 M NaOH to the ion solution with stirring and monitoring by a Benchtop pH meter (Orion 3 Star, Thermo Scientific, Beverly, MA, USA), which was calibrated using pH 4.0 and 7.0 buffer solutions prior to measurement.

### 2.4. Theoretical Calculations 

A stock solution of Pb^2+^ at a concentration of 500 ppm was prepared by dissolving the appropriate amount of Pb(NO_3_)_2_ in deionized water. All working solutions of the desired concentrations were obtained by further dilution with deionized water. A standard curve was established using 5-, 10-, 15-, and 20-ppm solutions (R^2^ = 0.9990). Sample concentrations were calculated according to the Beer–Lambert law with reference to the standard curve.

The equilibrium adsorption capacity and the percent removal of Pb^2+^ ions (Re%) were calculated using Equations (1) and (2),

(1) $$q_{t}=\frac{\left(C_{0}-C_{e}\right)V}{m}$$

(2) $$Re\%=\left(\frac{C_{0}-C_{e}}{C_{0}}\right)\times 100$$

where *q_t_*(mg/g) is the adsorption capacity of PPyAC4 for Pb^2+^ at time *t* (min) and *C*_0_ and *C_e_* (mg/L) are the liquid phase concentrations of Pb^2+^ before and after adsorption, respectively. *V* (L) and *m* (g) are the volume of the adsorption solution and the mass of the dry adsorbent used, respectively. 

#### 2.4.1. Adsorption Kinetics

The data obtained from the kinetic tests were fitted to the pseudo-first-order and pseudo-second-order models as stated by Lagergren-Svenska [35] and Ho-McKay [36], respectively. Pseudo-first-order Equation (3) assumes that the adsorption rate is based on the adsorption capacity. The pseudo-second-order model, Equation (4), is based on the assumption that the adsorption is controlled by the chemisorption mechanism, involving valency forces through electron sharing or transfer between the adsorbent and adsorbate [36,37].

(3) $$\log\left(q_{e}-q_{t}\right)=log(q_{e})-\frac{k_{1}t}{2.303}$$

(4) $$\frac{t}{q_{t}}=\frac{1}{k_{2}q_{e}^{2}}+\frac{t}{q_{e}}$$

where *q_t_* and *q_e_* (mg/g) are the amount of Pb^2+^ adsorbed per mass of adsorbent (g) at time *t* (min) and at equilibrium, respectively, and *k*_1_ (1/min) and *k*_2_ (mg/(g∙min)) are the rate constants of the pseudo-first-order and pseudo-second-order models, respectively.

The initial adsorption rate (*h*) can be calculated from the second-order rate constant as in Equation (5) [38].

(5) $$h=k_{2}q_{e}^{2}$$

#### 2.4.2. Adsorption Isotherms

The adsorption isotherm experiments were accomplished by shaking 25 mL of Pb^2+^ solutions of different concentrations (10–250 ppm) with 125 mg of PPyAC4 for 6 h, at pH 5.5. Two well-known models, Langmuir and Freundlich, were used to describe the isothermal characteristics of the adsorbent [39]. The linear forms of these models can be expressed as in Equations (6) and (7). However, the Langmuir isotherm [40] considers adsorption as a chemical phenomenon, assuming that adsorption occurs uniformly on the active sites of the surface and only a monolayer is formed. Freundlich, on the other hand, assumes a heterogeneous surface and an exponential distribution of active sites and their energies [2,41,42,43,44]. 

Langmuir linear model:

(6) $$\frac{C_{e}}{q_{e}}=\frac{C_{e}}{q_{m}}+\frac{1}{K_{L}q_{m}}$$

Freundlich linear model:

(7) $$logq_{e}=logK_{F}+\frac{1}{n}logC_{e}$$

where *C_e_* and *q_e_* have the same meaning as previously noted, *q_m_* (mg/g) is the maximum monolayer adsorption capacity of the adsorbent, *K_L_* (L/mg) is the Langmuir constant describing adsorption affinity for the adsorbent, *K_F_* (mg/g) (L/mg)^1/n^ is the Freundlich constant related to the multilayer adsorption capacity, and *n* is the heterogeneity factor, which represents the extent to which the adsorption depends on the equilibrium concentration. The essential feature of the Langmuir equation can be expressed in terms of a separation factor, *R_L_* (dimensionless), which is defined by the following equation.

(8) $$R_{L}=\frac{1}{1+K_{L}C_{e}}$$

### 2.5. Characterization

The surface morphologies of PPy and PPyAC4 were analyzed by scanning electron microscopy (SEM, JSM-6360 LV, JEOL, Tokyo, Japan) and transmission electron microscopy (TEM, JEM-1011, JEOL, Tokyo, Japan). Thermogravimetric analysis (TGA) was performed on a Mettler Toledo TGA/DSC 1 Star system (Columbus, OH, USA). The sample was heated from 25–800 °C at 25 °C/min under a nitrogen flow of 20 mL/min. X-ray diffraction (XRD) patterns were obtained using a Rigaku XtaLAB mini II benchtop X-Ray crystallography system (The Woodlands, TX, USA), with copper Kα radiation (λ = 1.5418 Å) and a scan speed of 3°/min. 

## 3. Results and Discussion 

### 3.1. PPyAC4 Properties 

#### 3.1.1. Morphological Analysis

**Figure 1:**
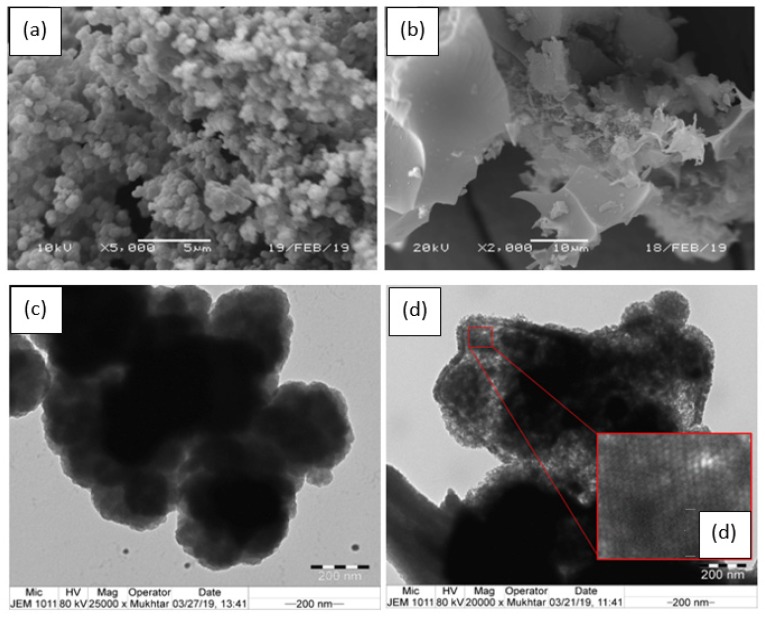
Scanning electron microscopy (SEM) (top row) and transition electron microscopy (TEM) (bottom row) images of PPy (**a**,**c**) and PPyAC4 (**b**,**d**). The insert (**d**) is a 6.5-times magnification of the specified area.

The surface morphology, thermal properties, and crystallinity of the carbonized PPy were analyzed using SEM, TEM, TGA, and XRD. The surface area and porosity, in addition to some other properties, were reported elsewhere (Table 1) [33]. The SEM and TEM images of the PPy used as the carbon precursor (Figure 1a,c) revealed its typical morphology as a cauliflower-like structure with an average diameter of 0.45 µm (reported as 0.59 µm [45] and ~1 µm [34]). This morphology appears to be due to the difficulty with which the dopant intercalates the disordered polymeric chain [46,47], as reported in the case of polythiophene films for which the role of dopant in the polymeric growth, uniformity, and extent of doping were investigated [48]. It has been concluded that the surface homogeneity and defects are related to the increase in the polymer thickness, the type of polymer, and the nature of the dopant. However, the spherical structure of PPy was destroyed through the carbonization process, resulting in a rough, rugged surface consisting of irregular, sharp corners and peel-like particles [34] with little evidence of porosity in the SEM images. The TEM image of PPyAC4 given in Figure 1d indicates the formation of graphene nanosheets; homogeneously-distributed micropores were seen with an average diameter of 12.5 nm, which is slightly higher than that indicated by BET data (2.3 nm). Furthermore, the TEM image depicts highly-abundant and symmetrical pores, with a shape previously determined to be long cylindrical capillaries [33]. The surface area and microporosity values corresponding to this activated carbon, prepared under the same temperature and activator conditions (KOH/PPy = 4) and a similar heating rate and time, have been reported previously [33,34]. The authors reported increased surface area and enhanced porosity characteristics with increased amounts of KOH. 

#### 3.1.2. XRD Analysis 

**Figure 2:**
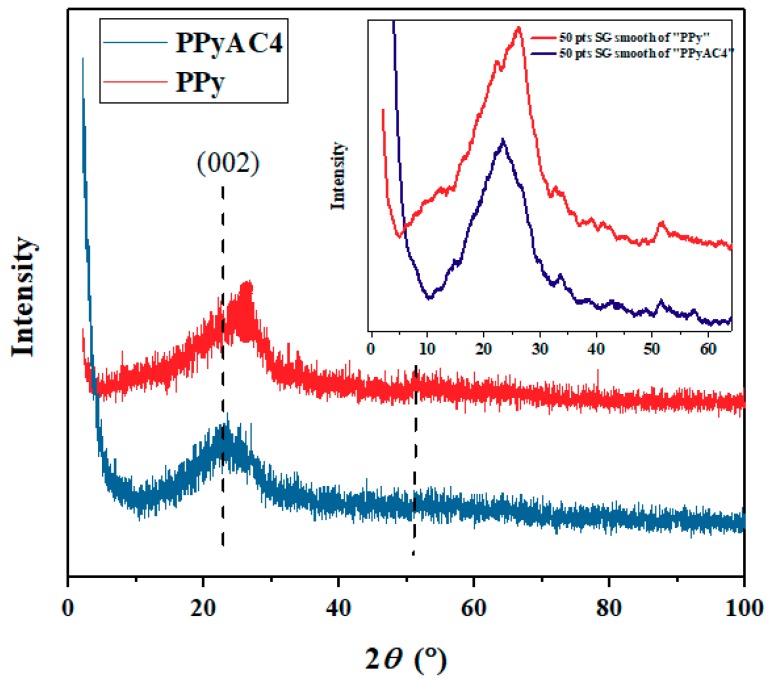
The X-ray diffraction patterns of PPy and PPyAC4. The inset is a 50 points (pts) smoothing of the data points according to a Savitzky–Golay (SG) method.

Figure 2 shows the X-ray diffraction (XRD) pattern of PPy and its corresponding activated carbon, PPyAC4. Typically, a broad reflection peak from 2θ = 10–30°, as well as an amorphous structure is observed for PPy. A similar pattern was observed for PPyAC4 in which the peak center was shifted to a lower 2θ value, i.e., from 26.4 to 23.7°, indicating that the interplanar spacing was increased. This could be due to the effect of the activating KOH, which could cause the sheets to be spaced further apart [45]. However, the analysis revealed that the PPyAC4 formed is amorphous in nature with short-disordered graphene sheets indicated by the broad peak at 2*θ* = 23.7° corresponding to the (002) plane [49]. In addition, the broad peak resulting from PPyAC4 was more symmetrical than the PPy peak, indicating a higher semi-crystalline structure than that of the substrate. A Savitzky–Golay (SG) smoothing method was applied to follow the peak center and the hidden peaks in the diffractogram, as seen in the insert of Figure 2. Thus, the peak shape became clearer, and shoulders could be identified; in addition, a weak peak at 2*θ* = 52° could be seen.

#### 3.1.3. Thermogravimetric Analysis 

**Figure 3:**
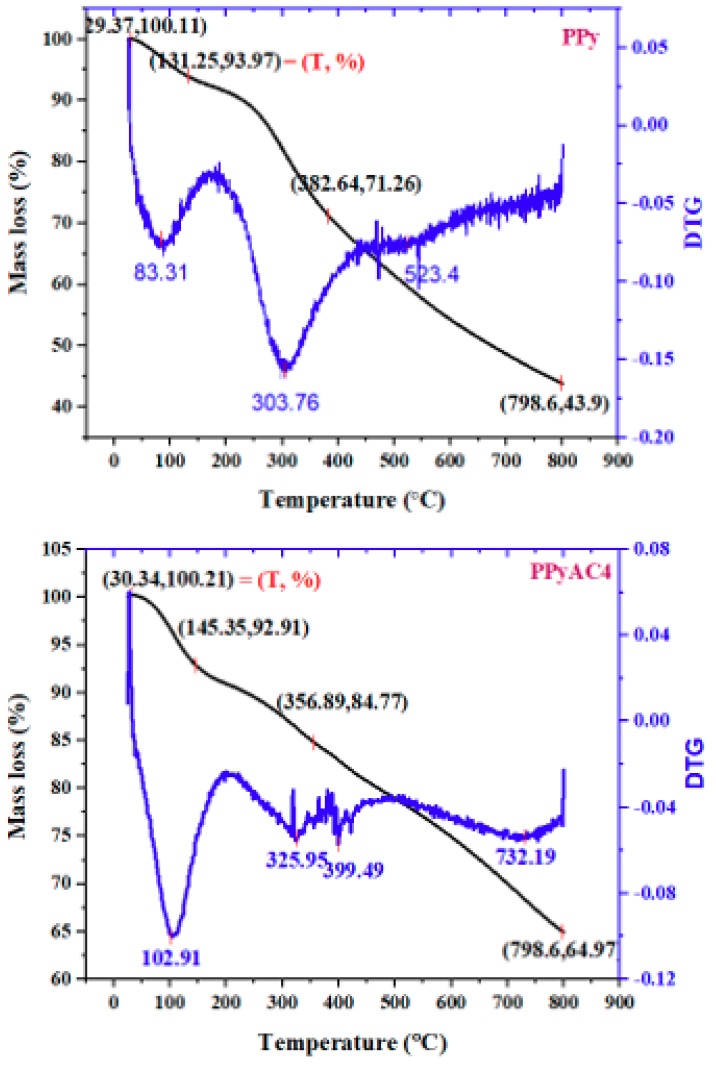
The thermogravimetric analysis (TGA) thermograms of PPy (top) and PPyAC4 (bottom) and their corresponding derivative-TGA (DTG) curves.

The thermal stability of PPy and PPyAC4 was assessed from the TGA and the derivative-TGA (DTG) curves (Figure 3). The DTG curves showed the mid-point decomposition temperature. The initial weight losses at about 83 °C (6%) and 103 °C (7%) for PPy and PPyAC4, respectively, could be attributed to the evaporation of water absorbed on the sample surfaces [50]. Thereafter, a gradual thermal decomposition of the sample backbone was noticed. The residual masses of PPy and PPyAC4 at 800 °C were found to be 44 and 65%, respectively, indicating a higher stability of the carbonaceous material.

### 3.2. Batch Method Adsorption Studies

All adsorption experiments were carried out at room temperature (i.e., 23 ± 2 °C) with a shaking speed of 130 rpm. The effect of several parameters, such as pH, adsorbate concentration, and adsorbent dose, on adsorption ability were studied. 

#### 3.2.1. Effect of Contact Time and Adsorption Kinetics 

**Figure 4:**
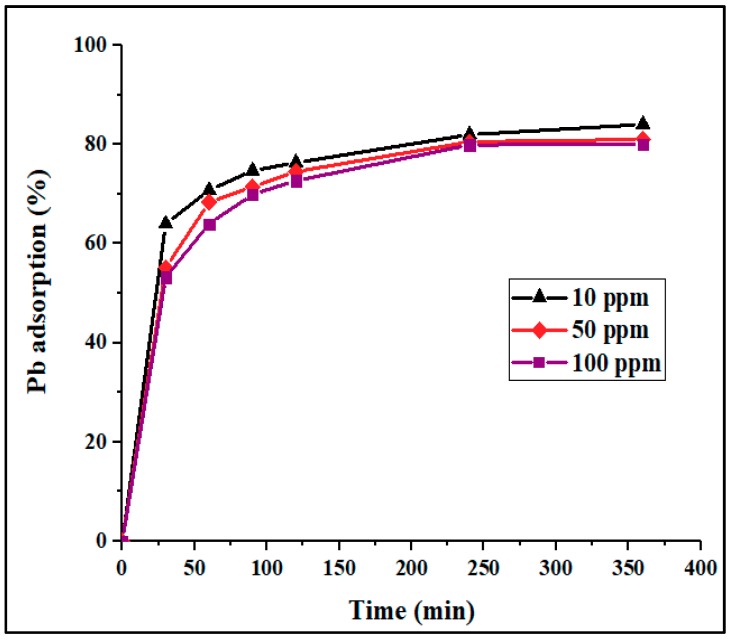
Adsorption percentage of Pb^2+^ onto PPyAC4 vs. time at three different concentrations: 10, 50, and 100 ppm. Experimental conditions: adsorbent dose 125 mg/25 mL, agitation speed 130 rpm, pH 5.5, temperature 23 ± 2 °C.

**Figure 5:**
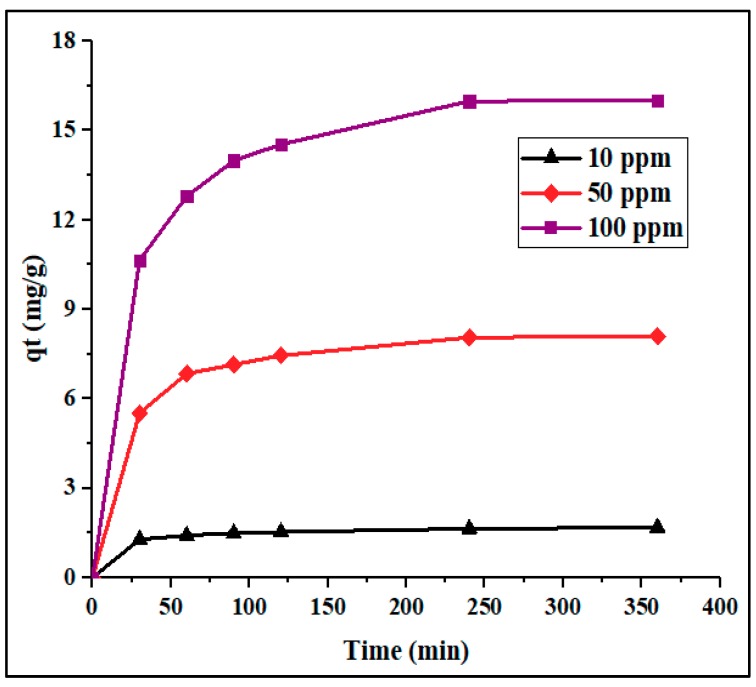
Adsorption capacity (mg/g) of PPyAC4 vs. time at three Pb^2+^ concentrations: 10, 50, and 100 ppm. Experimental conditions: adsorbent dose 125 mg/25 mL, agitation speed 130 rpm, pH 5.5, temperature 23 ± 2 °C.

Figure 4 shows the effect of contact time and Pb^2+^ concentration on adsorption percentage and capacity. In this figure, it can be seen that the Pb^2+^ adsorption was rapid in the first stage of adsorption, then slowly increased with time, becoming almost constant after 120 min. This phenomenon can be attributed to the high availability of vacant adsorption sites in the first stage of adsorption, followed by an increase in repulsive forces due to the presence of the adsorbed ions, making the remaining sites more difficult to access [37,41]. It was observed that the ion removal percentage was slightly reduced with an increase in initial concentration (Figure 4 and Figure 5). This behavior could be ascribed to the presence of a higher concentration of adsorbate per unit mass of adsorbent, which may restrict adsorption.

**Figure 6:**
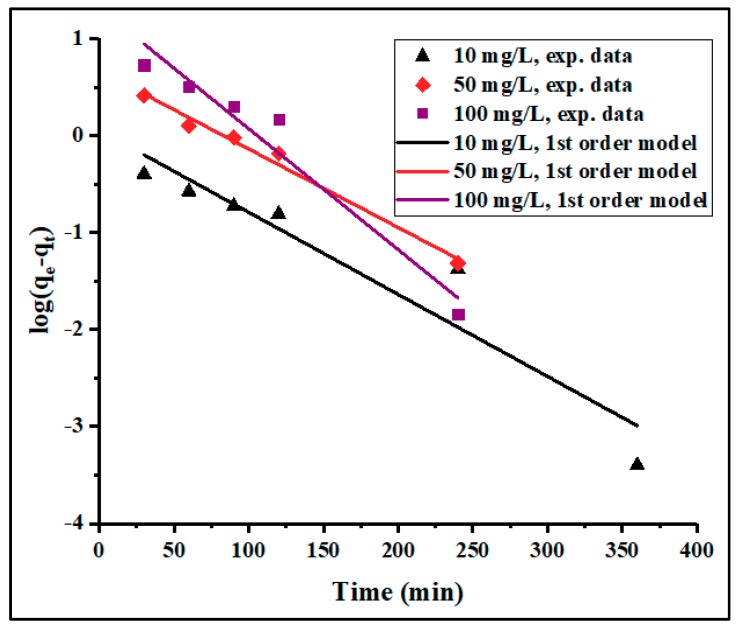
Pseudo-first-order kinetics of Pb^2+^ adsorption on PPyAC4 at 10, 50, and 100 ppm. Experimental conditions: adsorbent dose 125 mg/25 mL, agitation speed 130 rpm, pH 5.5, temperature 23 ± 2 °C. (exp = experiment).

**Figure 7:**
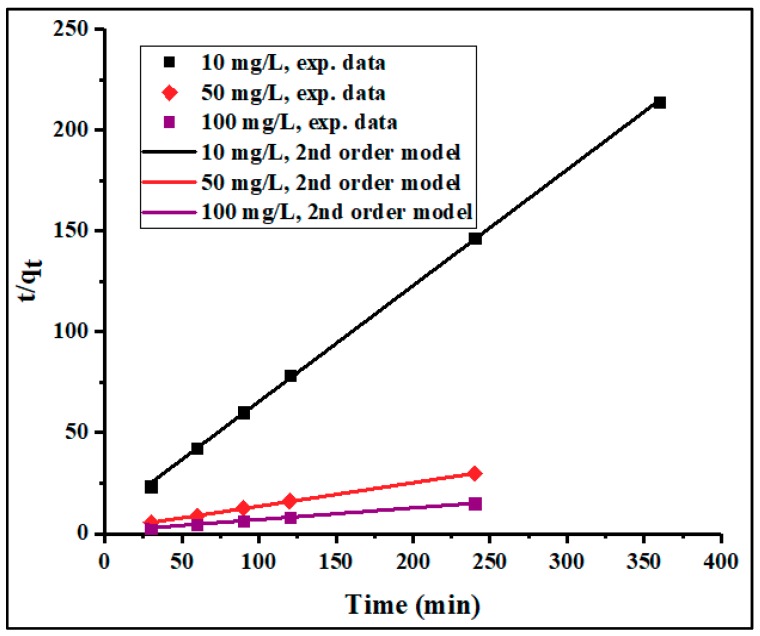
Pseudo-second-order kinetic of Pb^2+^ adsorption on PPyAC4 at 10, 50, and 100 ppm. Experimental conditions: adsorbent dose 125 mg/25 mL, agitation speed 130 rpm, pH 5.5, temperature 23 ± 2 °C. (exp = experiment).

To investigate the mechanism controlling the adsorption processes, the kinetic data collected in this study were fitted to the pseudo-first- and pseudo-second-order models. The linearized-integral equations, Equations (3) and (4), used to examine the two models are represented in Figure 6 and Figure 7. The calculated kinetic parameters are given in Table 2. Herein, the two models showed a good fit with the experimental data. However, the higher correlation coefficient (*R*^2^) values obtained from the pseudo-second-order (0.9997, 0.9997, and 0.9996) compared to those of the pseudo-first-order (0.9035, 0.9862, and 0.9507) kinetic model for the initial concentrations of 10, 50, and 100 mg/L, respectively, suggest that the adsorption more closely followed the pseudo-second-order kinetics. It was observed that the pseudo-second-order rate constant (*k*_2_) was inversely proportional to the initial concentration of Pb^2+^, indicating that the surface saturation was dependent on the initial ion concentration. Therefore, at low concentration, the binding sites took up the available ions quickly, but at higher concentration, the adsorption species needed to diffuse to the internal sites by intra-particle diffusion, resulting in reduced adsorption rate [51]. Furthermore, the effect of particle interaction, such as aggregation, may be the reason for the observed adsorption density decreases with the increase in adsorbent dose. In addition, the *q_e_* values calculated from the pseudo-second-order model were in agreement with the experimental values. Hence, the rate-limiting step of Pb^2+^ adsorption seemed to be controlled by a chemical adsorption process through the sharing or exchanging of electrons between adsorbent and adsorbate [22,52]. The initial adsorption rate (*h*) was also found to increase with increasing initial lead ion concentration. 

#### 3.2.2. Effect of pH 

**Figure 8:**
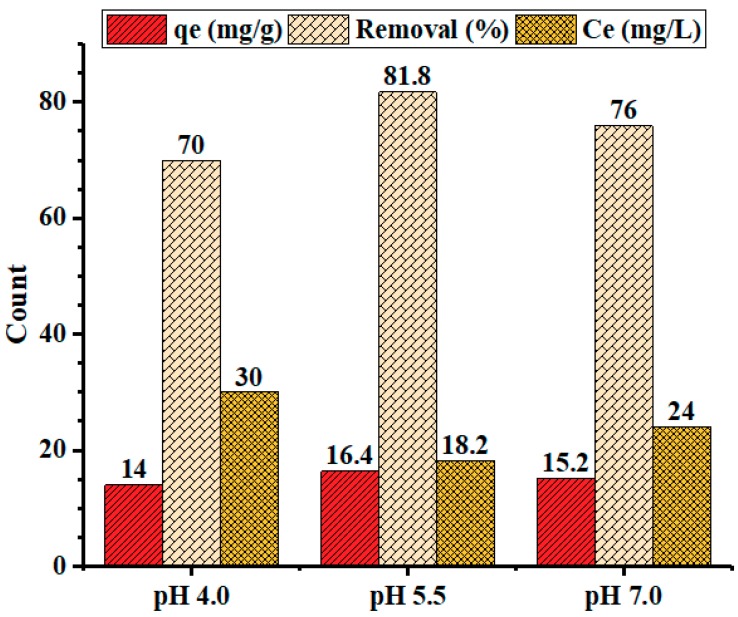
Graphical illustration of the effect of pH on the adsorption capacity and percent removal of lead by PPyAC4. Experimental conditions: adsorbent dose 125 mg/25 mL, agitation speed 130 rpm, temperature 23 ± 2 °C.

The adsorption of heavy metals is significantly affected by the pH of the solution, since it determines adsorbent properties such as surface charge, as well as the adsorbate speciation and degree of ionization in aqueous solutions. Adsorption studies were performed at various pH values; Figure 8 summarizes the adsorption data at pH 4.0, 5.5, and 7.0. The results indicated a higher adsorption percentage and capacity at pH 5.5 compared to those at the other examined pHs, a finding consistent with the literature [2,20,41]. Since the surface of the activated carbon is negative, electrostatic interactions with metal ions are expected. At a lower pH (e.g., 4.0), the adsorption of Pb^2+^ was low due to competition between protons and Pb^2+^ ions for the available adsorption sites [2]. As the pH increased from 4.0–5.5, there were fewer protonated active sites leaving more negatively-charged active sites available for electrostatic interaction with and thus adsorption of the positively-charged Pb^2+^ ions. The slight decrease in adsorption at pH > 5 could be attributed to the formation of soluble hydroxyl complexes, e.g., Pb(OH)^+^ and/or Pb(OH)_2_ [53]. Moreover, at pH > 7, lead hydroxide started to precipitate from the solution, making the adsorption studies impossible [54].

#### 3.2.3. Effect of Adsorbent Dosage

**Figure 9:**
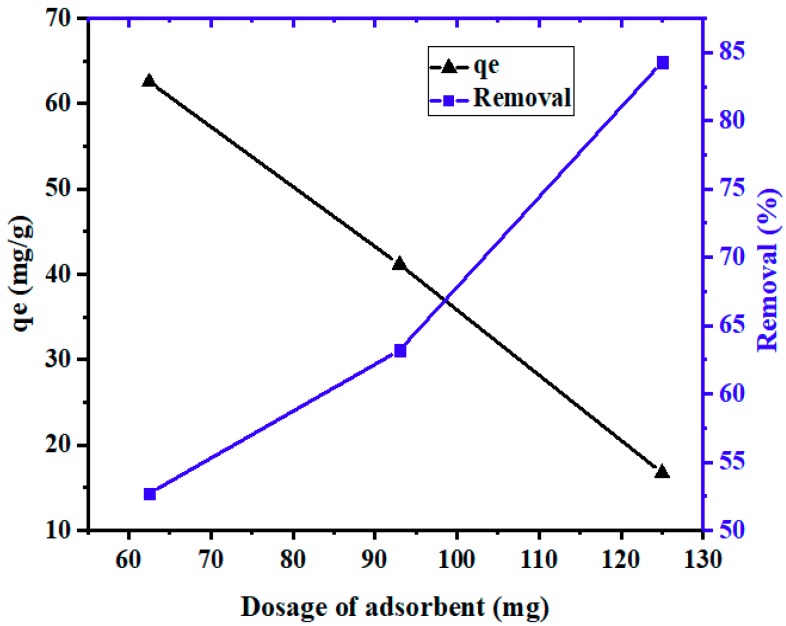
Effect of adsorbent dosage on the adsorption of Pb^2+^ by PPyAC4. Experimental conditions: adsorbent solution 25 mL, agitation speed 130 rpm, pH 5.5, temperature 23 ± 2 °C.

Figure 9 shows the effect of adsorbent dosage on the adsorption capacity of PPyAC4 for Pb^2+^ uptake. It is obvious that by increasing the adsorbent dosage, the adsorption capacity (q_e_, mg/g) decreased and the adsorption percentage increased as depicted in Figure 9. This might be due to the fact that all active sites were entirely exposed at lower doses, while only a fraction of the active sites were exposed at higher doses [22,55]. Thus, a higher adsorbent dosage may cause aggregation, which decreases the total surface area of adsorbent, leading to a decrease in adsorption [2,41,56].

### 3.3. Adsorption Isotherms 

#### 3.3.1. Langmuir Model

The Langmuir isotherm considers adsorption as a chemical phenomenon. This model assumes that all the available adsorption active sites are similar, the adsorbed species does not interact, and a monolayer is formed during adsorption [40]. The linear form of the Langmuir isotherm was applied for calculation of the corresponding parameters, given in Table 3. The results indicated that the adsorption capacity corresponding to the monolayer adsorption (*q_m_*) was 50 mg/g, and *K_L_*, the Langmuir constant related to the free energy of adsorption, was 0.02 (L/mg). When the essential parameter, *R_L_*, is between zero and one, this indicates favorable adsorption, while *R_L_* = 0 and *R_L_* ≥ 1 indicate irreversible and unfavorable adsorption isotherms, respectively. Thus, the obtained *R_L_* value of 0.450 suggested favorable adsorption of Pb^2+^ onto PPyAC4.

#### 3.3.2. Freundlich Isotherm

The Freundlich isotherm parameters were calculated and summarized in Table 3. According to the R^2^ value of both the Langmuir (0.989) and Freundlich (0.995) isotherms, it was concluded that the two models were reasonably suitable for describing adsorption. However, the Freundlich equation provided a better fit than the Langmuir equation. The value of *K_F_* indicated moderate affinity for Pb^2+^. Moreover, the Freundlich model is also characterized by the heterogeneity factor, 1/*n*. That is, 0.1 < 1/n < 1.0 indicates good adsorption of metal ions onto the adsorbent [53,57]. From the isotherm data (Table 3), it is clear that the Freundlich model well fit the adsorption, suggesting a chemical adsorption process, as indicated by the *n* value (1.276), the heterogeneity factor.

The adsorption capacity of other adsorbents for Pb^2+^ ion taken from the literature are given in Table 4 for comparison. The value of maximum adsorption capacity, *q*_m_, (mg/g) observed in this work was in good agreement with those found in literature. The differences of lead adsorption capacities are due to the differences in adsorbent properties such as structure, surface area, porosity, and functional groups. Additionally, the selection of the most suitable adsorbent depends on a number of factors such as cost-effectiveness, availability, and reusability. Adsorbents produced from agricultural and industrial wastes are generally less expensive than the synthesized or commercially-available activated carbons. However, the qualities and characteristics of activated carbons depend on both their precursors and activation methods. In Table 4, hazelnut husk, apricot stone, *Juniperus procera*, coconut shell, and *Polygonum orientale* Linn are agricultural and naturally-available products, utilized as low-cost materials for the preparation of activated carbon and investigated for the removal of lead ions from wastewater. In addition to their ability of regeneration, their values of *q_m_* vary between 13.05 and 98.39 (mg/g). On the other hand, acidified CNTs and PPy/oMWCNT composite (Table 4) are commercial products and are fabricated as adsorbents for lead ion removal. They have moderate adsorption capacities among the compared adsorbents, while their use may be costly. The adsorption capacity of the polypyrrole-based activated carbon (PPyAC4) was comparable. Although the PPyAC4 precursor was relatively inexpensive compared to CNTs, the facile preparation and activation methods can be extended to various polymer precursors, including low-cost, recyclable polymer waste.

## 4. Conclusions

To understand further the characteristics of the prepared polypyrrole-based activated carbon (PPyAC4), the resulting SEM, TEM, TGA, and XRD data were compared to those of the PPy substrate. PPyAC4 was obtained as an amorphous fine powder exhibiting encouraging surface properties including high surface area and porosity, indicating its potential for use as an adsorbent material for the removal of metal pollutants. The adsorption study demonstrated that PPyAC4 is an effective adsorbent for the removal of lead ions from their aqueous solutions. Adsorption equilibrium was attained within 4 h at the optimum pH of 5.5. The adsorption capacity at equilibrium, *q_e_*, was found to be greatly influenced by the concentration and pH of the solution, as well as the adsorbent dosage. The adsorption kinetics obeyed the pseudo-second-order model, suggesting that the rate-limiting step may be chemisorption rather than diffusion. Accordingly, the initial adsorption rate (*h*) was found to increase with increasing initial Pb^2+^ concentration, reaching 49.9 (mg/(g·min)) for the 100-ppm/25-mL solution. The adsorption equilibrium of Pb^2+^ was satisfactorily described by both the Langmuir and Freundlich equations along the studied concentration range, with better correlation to the Freundlich isotherm. The maximum adsorption (*q_m_*) calculated from the Langmuir equation was 50 mg/g.

## Figures and Tables

**Table 1:** Polypyrrole-based activated carbon (PPyAC4) properties: Brunauer–Emmett–Teller (BET) surface area, pore volume and diameter, and CO_2_ uptake capacity at 50 °C and 1.0 atm [33]. The pore diameter calculated from the transmission electron microscope (TEM) image is also included.

Sample	Surface Area (m^2^/g)	Average Pore Diameter (nm)		Pore Volume (cm^3^/g)	CO_2_ Uptake Capacity	
		BET	TEM		mg/g	mmol/g
PPyAC4	2871	2.3	12.5	0.054	49.95	1.14

**Table 2:** Kinetic parameters of pseudo-first- and pseudo-second-order equations for Pb^2+^ adsorption on PPyAC4 at C_o_ = 10, 50, and 100 ppm.

C_o_ (mg/L)	q_e_-exp (mg/g)	Pseudo-First Order			Pseudo-Second-Order			
		q_e_ (mg/g)	k_1_ (1/min)	R^2^	q_e_ (mg/g)	k_2_ (g/(mg·min))	R^2^	h (mg/(g·min))
10	1.682	1.138	1.170	0.9042	1.739	2.505	0.9997	7.575
50	8.104	4.710	1.119	0.9863	8.621	0.408	0.9997	30.323
100	16.01	20.845	1.723	0.9514	17.241	0.168	0.9996	49.938

**Table 3:** Langmuir and Freundlich isotherm parameters for Pb^2+^ adsorption on PPyAC4.

Adsorbent	Langmuir				Freundlich			
PPyAC4	*q*_m_ (mg/g)	*K_L_* (L/mg)	R^2^	*R_L_*	*1/n*	*n*	*K_F_* (mg/g)(L/mg)^1/n^	R^2^
	50	0.020	0.989	0.450	0.784	1.276	1.242	0.995

**Table 4:** Comparison of the adsorption capacity of various adsorbents for Pb^2+.^

Activated Carbon Source	*q*_m_ (mg/g)	Adsorption Conditions				Ref.
		pH	*T* (°C)	*C*_o_ (mg/L)	Adsorbent Dosage (g/L)	
Hazelnut husk (HH)	13.05	5.7	18	200	12.0	[58]
Acidified CNTs	17.44	5.0	-	10	-	[59]
Apricot stone	21.38	6.0	20	50	1.0	[53]
PPy/oMWCNT composite	26.32	6.0	25	10–100	1.0	[30]
*Juniperus procera*	30.3	4.6	25	50	8.0	[60]
Coconut shell	76.66	5.6	25	-	2.0	[61]
*Polygonum orientale* Linn	98.39	5.0	25	50–75	0.6	[42]
Polypyrrole-based AC	50.0	5.5	23	100	5.0	This work
